# P-1291. Racial disparities in Lyme disease among beneficiaries of US Medicaid and Medicare

**DOI:** 10.1093/ofid/ofae631.1472

**Published:** 2025-01-29

**Authors:** L Hannah Gould, Sarah J Willis, Christopher G Prener, Stephanie A Duench, Holly Yu, Jennifer Moisi, James H Stark

**Affiliations:** Pfizer Vaccines, New York, New York; Pfizer, Wakefield, Massachusetts; Pfizer, Wakefield, Massachusetts; Pfizer, Wakefield, Massachusetts; Pfizer Inc., Collegeville, Pennsylvania; Pfizer Vaccines, New York, New York; Pfizer Biopharma Group, Collegeville, Pennsylvania

## Abstract

**Background:**

Lyme disease (LD) is the most common vector-borne disease in the US. Manifestations range from a localized rash to neurologic, cardiac, and musculoskeletal conditions during disseminated stages. LD incidence is highest among White persons in the US, but evidence suggests that people from racial and ethnic minority groups experience more severe disease.

**Figure:**
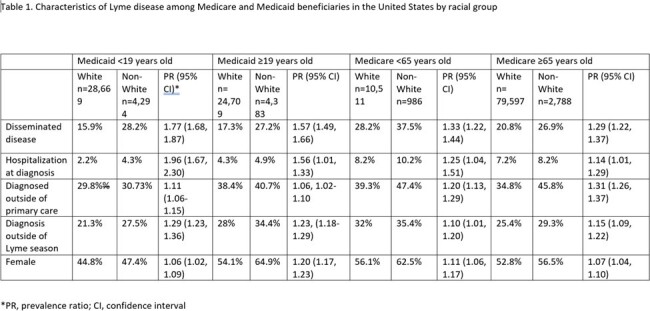

**Methods:**

We used a claims-based algorithm (≥ 1 LD ICD-10 code and appropriate antibiotics within 30 days) to identify LD cases among Medicaid (age < 19 and ≥ 19 years) and Medicare fee-for-service (age < 65 [persons with disabilities] and ≥ 65 years old) beneficiaries living in 16 high incidence states ( > 10 cases/100,000 population for ≥ 3 years) during 2016-2021. We calculated prevalence ratios for disseminated disease and hospitalization by racial group using White persons as the reference.

**Figure:**
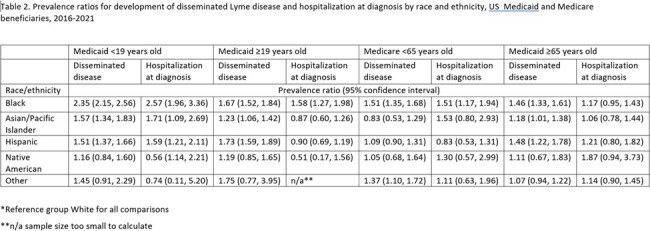

**Results:**

There were 62,055 LD cases identified among Medicaid beneficiaries and 90,882 among Medicare beneficiaries. In Medicaid, 85% of cases were in White persons, 6% in Hispanic, 4% in Black, 4% in Asian/Pacific Islander, and 1% in Native American persons. In Medicare, 96% of cases were in White persons, with 1% or less reported in other groups. Non-White persons were more likely to be hospitalized at diagnosis, diagnosed outside of primary care, diagnosed outside of the peak months for LD transmission, to be female, and to have disseminated disease (Table 1). Medicaid beneficiaries and Medicare beneficiaries ≥ 65 years old who identified as Black, Asian/Pacific Islander, or Hispanic had a higher likelihood of disseminated disease than White persons. Among Medicaid beneficiaries < 19 years old, those who identified as Black, Asian/Pacific Islander, or Hispanic had a higher likelihood of being hospitalized at diagnosis than those who identified as White. Black Medicaid beneficiaries ≥ 19 and Medicare beneficiaries < 65 also had a higher likelihood of hospitalization (Table 2).

**Conclusion:**

These data illustrate disparities in LD by race and ethnicity and suggest possible disparities by other characteristics including sex and, given the high rates of disseminated disease in Medicare beneficiaries < 65 years old, disability. Equitable interventions are needed to reduce disparities in LD recognition and severity.

**Disclosures:**

**L. Hannah Gould, PhD, MS, MBA**, Pfizer: Employee|Pfizer: Stocks/Bonds (Public Company) **Sarah J. Willis, PhD, MPH**, Pfizer, Inc.: Employment|Pfizer, Inc.: Stocks/Bonds (Private Company) **Christopher G. Prener, PhD**, Pfizer, Inc.: Employee|Pfizer, Inc.: Stocks/Bonds (Public Company) **Stephanie A. Duench, PhD**, Pfizer Inc.: Employee|Pfizer Inc.: Stocks/Bonds (Public Company) **Holly Yu, MSPH**, Pfizer: Employee|Pfizer: Stocks/Bonds (Public Company) **Jennifer Moisi, PhD**, Pfizer: Employee|Pfizer: Stocks/Bonds (Public Company) **James H. Stark, PhD**, Pfizer: Employee|Pfizer: Stocks/Bonds (Public Company)

